# Sex-specific IL-6-associated signaling activation in ozone-induced lung inflammation

**DOI:** 10.1186/s13293-016-0069-7

**Published:** 2016-03-05

**Authors:** Vikas Mishra, Susan L. DiAngelo, Patricia Silveyra

**Affiliations:** Department of Pediatrics, The Pennsylvania State University College of Medicine, 500 University Drive, H085, Hershey, PA 17033 USA; Department of Biochemistry and Molecular Biology, The Pennsylvania State University College of Medicine, Hershey, PA 17033 USA

**Keywords:** Inflammation, Oxidative stress, Lung disease, Gender differences, Estrous cycle

## Abstract

**Background:**

Acute ozone (O_3_) exposure has known deleterious effects on the respiratory system and has been linked with respiratory disease and infection. Inflammatory lung disease induced by air pollution has demonstrated greater severity and poorer prognosis in women vs. men. Both severe damage to the bronchial-alveolar epithelium and malfunctioning of bronchial-blood barrier have been largely attributed to the pathobiology of O_3_-induced inflammatory response, but the associated mechanisms in the male and female lung remain unknown.

**Methods:**

Here, we investigated sex-based differential regulation of lung interleukin-6 (IL-6) and its downstream signaling pathways JAK2/STAT3 and AKT1/NF-κB in response to O_3_ exposure in a mouse model. We exposed male and female mice (in different stages of the estrous cycle) to 2 ppm of O_3_ or filtered air (FA) for 3 h, and we harvested lung tissue for protein expression analysis by Western blot.

**Results:**

We found significant up-regulation of IL-6 and IL-6R in females and IL-6 in males in response to O_3_ vs. FA. Ozone exposure induced a significant increase in STAT3-Y705 phosphorylation in both females and males. Males exposed to O_3_ had decreased levels of JAK2, but increased JAK2 (Y1007+Y1008) phosphorylation, while females exposed to O_3_ showed significant up-regulation of both proteins. Both NF-κB (p105/p50) and AKT1 protein levels were significantly increased only in females exposed to O_3_. In addition, females exposed to O_3_ during proestrus displayed increased expression of selected genes when compared to females exposed to O_3_ in other estrous cycle stages.

**Conclusions:**

Together, our observations indicate a sex-based and estrous cycle-dependent differential lung inflammatory response to O_3_ and involvement of two converging JAK2/STAT3 and AKT1/NF-κB pathways. To our knowledge, this is the first study specifically addressing the impact of the estrous cycle in O_3_-associated lung inflammatory pathways.

**Electronic supplementary material:**

The online version of this article (doi:10.1186/s13293-016-0069-7) contains supplementary material, which is available to authorized users.

## Background

Ground-level ozone (O_3_) is a photochemical air pollutant and a powerful oxidant formed by the action of sunlight on nitrogen oxides and reactive hydrocarbons, both of which are emitted by motor vehicles and industrial sources [1]. Exposure to O_3_ even within the safe concentration range as per the standard definition by Environmental Protection Agency can affect breathing and lung function and has deleterious effects on pulmonary innate immunity [2–4]. Acute O_3_ exposure is toxic to the respiratory system and has been linked with respiratory tract infections, asthma, chronic obstructive pulmonary disease, cystic fibrosis, lung cancer, and cardiovascular disease, with relatively poor prognosis and higher mortality in women than in men [5–11]. Studies point to gender-based differences in incidence, risk, severity, and pathology of certain environmental lung diseases in women as compared to men [12–14]. Growing evidence indicated that females are more susceptible to the toxic effects of tobacco [15, 16], have worse respiratory symptoms [17, 18], and higher airway responsiveness [19] compared to males. Several studies have proposed circulating hormone levels as potential regulatory factors of immune responses in the female [20–23]. However, the mechanisms associated with the differential lung disease outcomes in men and women are still poorly understood.

Acute O_3_ exposure and manifestation of clinical respiratory symptoms can primarily be attributed to formation of cytotoxic products and acute cellular injury through oxidative stress, causing biochemical and physiological changes in the lung epithelium. These are mediated by an increased production of reactive oxygen species, accumulation of oxidized biomolecules and activation of inflammatory processes, both locally and systemically [24, 25]. The pathologic response of acute O_3_ exposure associated oxidative stress is yet to be completely elucidated. However, investigations indicate a crucial involvement of O_3_-associated damage to the bronchial-alveolar epithelium as a potential mechanism [26, 27]. Malfunction of the bronchial-alveolar epithelium and bronchial-blood barrier due to loss of integrity of tight junctions may increase immune cell infiltration and inflammatory response. Predominantly, studies have indicated involvement of two groups of markers in O_3_ pathobiology, the free arachidonic acid and its metabolites [28, 29] and cytokines [30–32]. Cytokines have been implicated as potential mediators of lung oxidative injury [33, 34]. Recent studies have shown induction of acute-phase cytokines including IL-1, IL-2, IL-6, IL-8, and TNF-α, as well as the neutrophil chemotactic factors keratinocyte-derived chemokine (KC), MIP-2, and LPS-induced CXC chemokine (LIX), following O_3_ exposures [30, 35–39]. Many of these secreted factors are recognized downstream products of activation of the innate immune system. These data suggest that downstream activation of pro-inflammatory factors play an important role in response to ambient O_3_; however, the stimulus leading to activation of these pro-inflammatory factors and their cross-talk remains poorly understood.

Previously, we have reported that exposure of O_3_ significantly decreased survival of male and female mice after bacterial infection [27]. Infected females exposed to O_3_ had more pronounced lung inflammation and higher mortality rates compared to males [29]. Our additional work demonstrated that gonadal hormones are responsible for the observed sex differences, indicating that both sex and air pollution may alter the effectiveness of lung host defense [28]. Recently, we have showed sex differences in the messenger RNA (mRNA) expression of cytokines, chemokines, and oxidative stress-related enzymes in the lungs of filtered air (FA, control) and O_3_ exposed animals [39].

The cytokine IL-6 demonstrates pleiotropy and functional redundancy. We have previously demonstrated that neutrophil-attracting chemokines (Ccl20, Cxcl5, and Cxcl2) and pro-inflammatory cytokine IL-6 mRNAs are most affected by ozone inhalation in both males and females, where females had significantly higher expression levels compared to the males [39]. It has also been reported that ozone increases airway neutrophil recruitment, which contributes to acute lung injury and hyper-reactivity and promotes inflammatory lung diseases [40–43]. The ability of alveolar macrophages to express IL-6 after ozone exposure and importance of IL-6 in pulmonary neutrophil recruitment following ozone exposure raises an important question, whether IL-6 and its sequential downstream pathways exhibit any sex differences and whether those differences can be accounted for higher susceptibility and severity of lung diseases in females. In an effort to study the overall effects of O_3_ in mediating acute inflammation and oxidative stress in the lungs of males and females, the present study evaluates a possible role of JAK2/STAT3 and AKT1/NF-κB signaling in relation to IL-6 and IL-6R response and the effects of the female estrous cycle in O_3_-induced lung inflammation.

At the cellular level, an inflammatory event is primarily characterized by an initial influx of neutrophils, which is subsequently replaced by inflammatory monocytes and T cells. IL-6 has been shown to be a key player in both acute and chronic inflammation and can dictate the profile of leukocyte recruitment during the inflammatory response via selective regulation of inflammatory chemokines/cytokines and apoptotic events. This paper extends our previous work and describes the possible role of IL-6R-associated signaling pathways in the inflammatory response to O_3_ in the male and female lung, as well as the contributions of the female estrous cycle to this regulation. Our results indicate a sex-based differential, and estrous cycle stage-dependent lung immune response to O_3_, and involvement of two converging JAK2/STAT3 and AKT1/NF-κB pathways in O_3_-associated lung inflammation. To our knowledge, this is the first study specifically addressing the impact of the female reproductive cycle in lung inflammatory pathways associated with O_3_ exposure.

## Methods

### Animals

Adult male and female mice (8 weeks of age) from the C57BL/6 background were purchased from JAX laboratories (Bar Harbor, ME) and housed and maintained in a 12/12 h light/dark cycle, with food and water available ad libitum. The Pennsylvania State University College of Medicine Institutional Animal Care and Use Committee approved all procedures.

### Assessment of estrous cycle stage in female mice

Assessment of estrous cycle stages in females was performed by the analysis of vaginal secretions for at least three consecutive cycles, as described previously [44]. Briefly, mice were restrained, and their vaginas were flushed daily with PBS. For vaginal cytology, a smear of vaginal flush was prepared and observed unstained under light microscope at ×10 and ×40 objectives. Determination of the estrous cycle stage was decided on the proportion among three cell types: nucleated epithelial cells, leucocytes, and cornified cells, where (A) proestrus was defined for a smear containing predominantly nucleated epithelial cells; (B) estrus was defined for a smear with majority of anucleated cornified cells; (C) metestrus, for smears consisting of the three types of cells; and (D) diestrus, in smears consisting predominantly of leucocytes. Animals that did not cycle regularly were excluded from the experiment.

### Ozone exposure

Male and female mice (at different stages of estrous cycle) were exposed to 2 ppm of O_3_ or FA (control) for 3 h, in different chambers as described previously [22, 45, 46]. Briefly, mice were exposed to ozone or to FA at the same time in separate chambers. Each chamber consisted of a 3.7-L closed glass vessel into which glass containers with wire mesh tops were placed. The temperature was maintained at 25 °C, humidity was set to 50 %, and the flow rate was 15 L/min through each (FA and ozone) chamber. Air flow and ozone content were continually monitored. All FA and ozone exposures were conducted in parallel. Animals were sacrificed 4 h after exposure, and total lung tissue was collected for Western blot experiments (*n* = 22 animals per group). Blood was also collected in female mice for serum hormone determinations.

### Assessment of serum hormone levels in female mice

Serum levels of progesterone were determined by ELISA (cat #MBS266675, MyBioSource, San Diego, CA) at the Penn State Hershey core endocrine laboratory. Serum levels of luteinizing hormone (MBS041300, MyBioSource, San Diego, CA) were also determined by ELISA.

### Protein extraction and Western blot

RIPA buffer (Thermo, Rockford, IL) was used to extract protein from pulverized lung tissues, following the manufacturer’s protocol. Protein concentration was determined by BCA assay (Thermo, Rockford, IL), and 20 μg were used for Western blot analysis with the respective antibodies. For densitometric quantitation of Western blots, images were digitized by using a BioRad GS800 calibrated densitometer and analyzed on BioRad Quantity One software (Penn State Hershey Core Facility). Quantification for the difference in the expression was assessed following normalization to GAPDH.

### Primary and secondary antibodies

Antibodies to IL-6 (AB6672) and IL-6R (AB83053), STAT3-unphosphorylated (AB68153), STAT3 Serine 727 phosphorylation (AB86430), STAT3 Tyrosine 705 phosphorylation (AB76315), JAK2-unphosphorylated (AB98031 and AB108596), JAK2 phosphorylated-Y1007+Y1008 (AB68268), NF-κB-p105/p50 (AB32360), AKT1 (AB32505), and GAPDH (AB9485) were obtained from Abcam (Cambridge, MA). All HRP-conjugated secondary antibodies were from Invitrogen.

### Statistical analysis

A total of 88 animals were used in the study with 22 animals in each study arm comprising of male filter air, male ozone, female filter air, and female ozone. Based on the assessment of estrous cycle stages, females (*n* = 44) were further sub-grouped in non-proestrus female filter air, non-proestrus female ozone, proestrus female filter air, and proestrus female ozone categories. The differences between O_3_- and FA-exposed animals were first compared by Kruskal-Wallis analysis of variance of the densitometric data of Western blot experiments. The values are depicted as mean with SD. *p* ≤ 0.05 were considered significant, where, *p ≤ 0.05, **p ≤ 0.01, ***p ≤ 0.001 and ****p ≤ 0.0001 are the levels of statistical significance compared to controls. Statistical analyses were performed using GraphPad Prism version 6.00 for Mac OS X, GraphPad Software (La Jolla California USA, www.GraphPad.com). To further ascertain the effect of sex and female estrous cycle stages, a two-way ANOVA analysis was performed using SPSS version 22.0 (IBM Corp. Released 2013. IBM SPSS Statistics for Windows, Version 22.0. Armonk, NY: IBM Corp.) on IL6, IL6R, STAT3 (unphosphorylated), STAT3 Serine 727 and Tyrosine 705 phosphorylation, JAK2 and JAK2 (Y1007+Y1008) phosphorylation, NF-κB (p105/p50), and AKT1 expression with filter air and ozone exposure. Residual analysis was performed to test for the assumptions of the two-way ANOVA. Outliers were assessed by inspection of a boxplot, normality was assessed using Shapiro-Wilk’s normality test for each cell of the design, and homogeneity of variances was assessed by Levene’s test. Analyses of simple main effects (the effect of one factor at each level of the other factor) for sex and estrous cycle stage compared to the type of exposure were performed with statistical significance receiving a Bonferroni adjustment and being accepted at the *p* < 0.025 level. For the interpretation of significant interactions, it was also investigated if the interaction effects were ordinal or disordinal.

## Results

### Ozone-associated lung inflammation and up-regulation of IL-6 and IL-6R expression

**Fig. 1:**
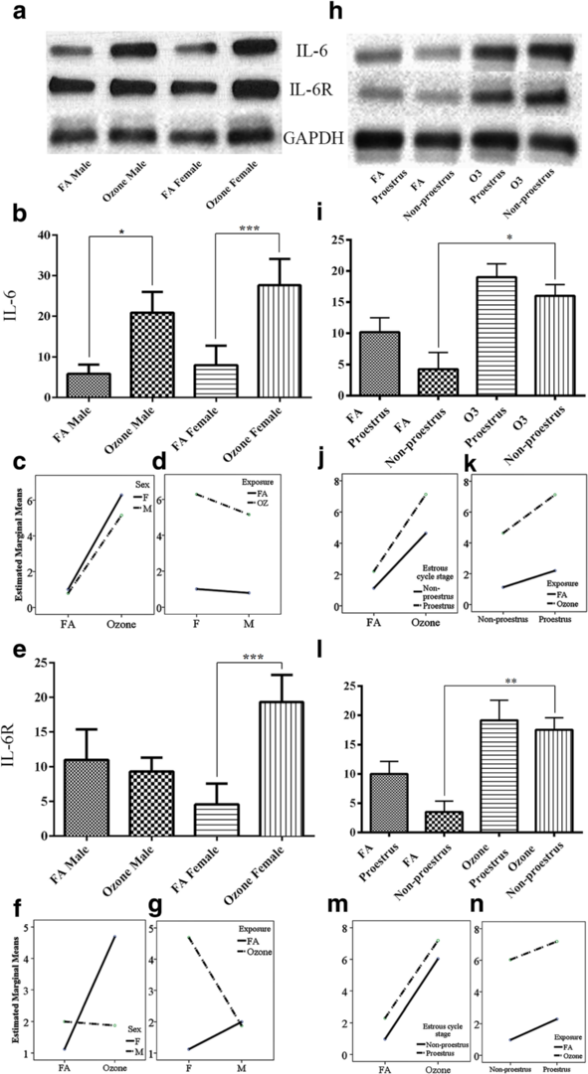
IL6 and IL6R expression and effect of ozone exposure. *Left panels*: **a** Representative Western blot images of IL6 and IL6R expression in males and females with filter air and O_3_ exposure; univariate analysis of IL6 (**b**) and IL6R (**e**), expression in males and females, with FA and O_3_ exposure; two-way ANOVA interaction effect of sex (**c**) and exposure (**d**), for IL6 expression and sex (**f**) and exposure (**g**), for IL6R expression. *Right panels*: **h** Representative Western blot images of IL6 and IL6R expression in estrous cycle stages of females, with filter air and O_3_ exposure; univariate analysis of IL6 (**i**) and IL6R (**l**), expression in estrous cycle stages of females, with FA and O_3_ exposure. Two-way ANOVA interaction effect of exposure (**j**) and estrous cycle stages (**k**), for IL6 expression and exposure (**m**) and estrous cycle stages (**n**), for IL6R expression. Univariate analysis data expressed as Ranks-Kruskal-Wallis test of densitometric analysis; the values are depicted as mean with SD, where *p ≤ 0.05, **p ≤ 0.01, and ***p ≤ 0.001 are the levels of statistical significance compared to controls (*n* = 6–8 per group). Two-way ANOVA for IL6 and IL6R analysis is given in Tables 1 and 3, respectively

With one-way analysis of variance, ozone exposure resulted in a significant increase in the expression levels of IL-6 in both male and female mice compared to the matched controls exposed to FA (Fig. 1a, b, Additional file 1: Figure S1-a). However, the expression of IL-6 was significantly higher in females in comparison to males exposed to O_3_ (Fig. 1a, b). A two-way ANOVA on the sex and ozone/filter air exposure showed statistically significant interaction *F* (1, 52) = 6.55, *p* = 0.013, partial η2 = 0.112. An analysis of simple main effects for sex and exposure type with Bonferroni adjustment revealed statistically significant difference in IL6 expression score between filter air and ozone exposure. Ozone-exposed females had a significant increase in the IL6 score of 0.566 (95 % CI, 0.313 to 0.819) points compared to the ozone-exposed males, *F* (1, 52) = 20.11, *p* = <0.0005, partial η2 = 0.279 (Table 1). Similarly, females with ozone exposure had significant increase in the IL6 score of 2.641 (95 % CI, 2.38 to 2.89) points compared to the females exposed to the filter air, *F* (1, 52) = 438.0, *p* = <0.0005, partial η2 = 0.894 (Table 1). Interaction effect of sex and exposure for IL6 expression is given in Fig. 1c, d.

**Table 1:** Two-way ANOVA for univariate and pairwise comparisons between gender, estrous cycle stages, and exposure for IL6 expression

Univariate tests: dependent variable: IL6							
			*df*	*F*	Sig.	Partial η2	
Analysis of gender and exposure	Exposure	FA	1, 52	0.751	0.390	0.014	
		Ozone	1, 52	20.113	<0.0005	0.279	
	Sex	F	1, 52	438.088	<0.0005	0.894	
		M	1, 52	299.716	<0.0005	0.852	
Analysis of female estrous cycle stages and exposure	Estrous cycle stage	Non-proestrus	1, 24	55.368	<0.0005	0.698	
		Proestrus	1, 24	108.679	<0.0005	0.819	
	Exposure	FA	1, 24	5.228	0.031	0.179	
		Ozone	1, 24	27.777	<0.0005	0.536	
Pairwise comparisons: dependent variable: IL6							
			(I) Sex	(J) Sex	Mean difference (I–J)	95 % CI for difference	
						Lower bound	Upper bound
Analysis of gender and exposure	Exposure	FA	F	M	0.109	0.144	0.363
		Ozone	F	M	0.566	0.313	0.819
	Sex	F	Ozone	FA	2.641	2.388	2.895
		M	Ozone	FA	2.185	1.932	2.438
Analysis of female estrous cycle stages and exposure	Estrous cycle stage	Non-proestrus	Ozone	FA	1.758	1.270	2.245
		Proestrus	Ozone	FA	2.462	1.975	2.950
	Exposure	FA	Proestrus	Non-proestrus	.540	0.053	1.028
		Ozone	Proestrus	Non-proestrus	1.245	0.757	1.732

To evaluate the contributions of female hormones to this regulation, we further investigated IL-6 levels in females exposed to O_3_ or FA at different stages of the estrous cycle. Serum hormone measurements performed at the time of sample collection (between 6:00 pm and 7:00 pm) indicated higher levels of luteinizing hormone and progesterone in proestrus vs. other estrous cycle stages (Table 2), as previously described [47]. Thus, for the purpose of this work, we performed comparisons of lung protein expression levels in proestrus vs. the rest of the days combined. With one-way analysis of variance, we found that exposure to O_3_ on the day of proestrus had a slightly higher but non-significantly different effect on lung IL-6 expression than exposure in non-proestrus cycle stages (Fig. 1h, i); however statistically, the O_3_ exposed non-proestrus cycle stage had a significant increase in the expression due to much lower IL-6 levels in the matched FA-exposed group. A two-way ANOVA examining the effects of estrous cycle stages and filter air/ozone exposure on the IL6 expression, showed statistically significant interaction *F* (1, 24) = 4.45, *p* = 0.045, partial η2 = 0.156. An analysis of simple main effects for estrous cycle stages and exposure type with Bonferroni adjustment revealed statistically significant difference in IL6 expression score. Proestrus females exposed to ozone had a significant increase in the IL6 score of 2.462 (95 % CI, 1.97 to 2.95) points compared to the filtered air-exposed proestrus females, *F* (1, 24) = 108.67, *p* = <0.0005, partial η2 = 0.819 (Table 1). Similarly, ozone-exposed proestrus females had a significant increase in the IL6 score of 1.245 (95 % CI, 0.757 to 1.732) points compared to the non-proestrus females exposed to ozone, *F* (1, 24) = 27.77, *p* = <0.0005, partial η2 = 0.536 (Table 1). Filter air/ozone exposure on each sex alone and estrous cycle type alone in relation to the IL6 is given in Table 1. Interaction effect of exposure and estrous cycle stages for IL6 expression is given in Fig. 1j, k.

**Table 2:** Serum hormone levels in female mice

	Proestrus	Estrus	Metestrus	Diestrus	Non-proestrus combined
LH (mIU/ml)	11.0 ± 0.9	3.4 ± 0.4	4.4 ± 0.5	3.8 ± 0.5	3.9 ± 0.3*
Progesterone (ng/ml)	5.6 ± 1.3	2.3 ± 0.4	2.1 ± 0.3	2.0 ± 0.2	2.2 ± 0.2*

*p < 0.05 vs. proestrus

Levels of IL-6R exerted an overall increase (Additional file 1: Figure S1-b) and a marked difference in expression between male and female mice exposed to O_3_ or FA (Fig. 1a, e). With one-way analysis of variance, the basal levels of IL-6R in female mice exposed to FA were lower compared to male mice. With O_3_ exposure, we found little to no change in the expression levels of IL-6R in male mice, while a very significant increase in female mice (Fig. 1a, e). A two-way ANOVA on the sex and ozone/filter air exposure showed statistically significant interaction *F* (1, 52) = 37.94, *p* = <0.0005, partial η2 = 0.422. An analysis of simple main effects for sex and exposure type with Bonferroni adjustment revealed statistically significant difference in IL6R expression score between filter air and ozone exposure. Ozone-exposed females had a significant increase in the IL6R score of 1.409 (95 % CI, 0.984 to 1.835) points compared to the ozone-exposed males, *F* (1, 52) = 44.13, *p* = <0.0005, partial η2 = 0.459 (Table 3). Similarly, females with ozone exposure had a significant increase in the IL6R score of 1.785 (95 % CI, 1.36 to 2.211) points compared to the females exposed to the filter air, *F* (1, 52) = 70.84, *p* = <0.0005, partial η2 = 0.577 (Table 3). Interaction effect of sex and exposure for IL6R expression is given in Fig. 1f, g.

**Table 3:** Two-way ANOVA for univariate and pairwise comparisons between gender, estrous cycle stages, and exposure for IL6R expression

Univariate tests: dependent variable: IL6R							
			*df*	*F*	Sig.	Partial η2	
Analysis of gender and exposure	Exposure	FA	1, 52	4.275	0.044	0.076	
		Ozone	1, 52	44.137	<0.0005	0.459	
	Sex	F	1, 52	70.840	<0.0005	0.577	
		M	1, 52	0.087	0.770	0.002	
Analysis of female estrous cycle stages and exposure	Estrous cycle stage	Non-proestrus	1, 24	150.946	<0.0005	0.863	
		Proestrus	1, 24	141.524	<0.0005	0.855	
	Exposure	FA	1, 24	10.240	0.004	0.299	
		Ozone	1, 24	7.898	0.010	0.248	
Pairwise comparisons: dependent variable: IL6R							
			(I) Sex	(J) Sex	Mean difference (I–J)	95 % CI for difference	
						Lower bound	Upper bound
Analysis of gender and exposure	Exposure	FA	M	F	0.439	0.013	0.864
		Ozone	F	M	1.409	0.984	1.835
	Sex	F	Ozone	FA	1.785	1.360	2.211
		M	FA	Ozone	0.062	0.363	0.488
Analysis of female estrous cycle stages and exposure	Estrous cycle stage	Non-proestrus	Ozone	FA	2.529	2.104	2.954
		Proestrus	Ozone	FA	2.449	2.024	2.874
	Exposure	FA	Proestrus	Non-proestrus	0.659	0.234	1.084
		Ozone	Proestrus	Non-proestrus	0.579	0.154	1.003

In concordance with the IL-6 expression, one-way analysis of variance of female mice exposed to O_3_ at different estrous cycle stages showed a slightly higher, but not significant, expression of IL-6R in proestrus compared to non-proestrus (Fig. 1h, l). A two-way ANOVA examining the effects of estrous cycle stages and filter air/ozone exposure on the IL6R expression, showed statistically insignificant interaction *F* (1, 24) = 0.076, *p* = 0.785, partial η2 = 0.003. However, an analysis of simple main effects for estrous cycle stages and exposure type with Bonferroni adjustment revealed statistically significant difference in IL6R expression score. Non-proestrus female exposed to ozone had a significant increase in the IL6R score of 2.592 (95 % CI, 2.104 to 2.954) points compared to the filtered air-exposed non-proestrus female, *F* (1, 24) = 150.94, *p* = <0.0005, partial η2 = 0.863 (Table 3). Likewise, filtered air-exposed proestrus females had a significant increase in the IL6R score of 0.659 (95 % CI, 0.234 to 1.084) points compared to the non-proestrus females exposed to the filter air, *F* (1, 24) = 10.240, *p* = 0.004, partial η2 = 0.299 (Table 3). Filter air/ozone exposure on each sex alone and estrous cycle type alone in relation to the IL6R is given in Table 3. Interaction effect of exposure and estrous cycle stages for IL6R expression is given in Fig. 1m, n.

### Ozone-associated lung inflammation and expression of STAT3 unphosphorylated and STAT3 Serine 727 and Tyrosine 705 phosphorylation

In order to establish the possible role of STAT3 in relation to the IL-6 and IL-6R expression, we further investigated the expression levels of STAT3 unphosphorylated, as well as STAT3 Serine 727 (p-S727) and Tyrosine 705 (p-Y705) phosphorylation in mice exposed to O_3_ or FA. The expression levels were further compared between males and females, and the impact of exposure in different female estrous cycle stages was assessed.

**Fig. 2:**
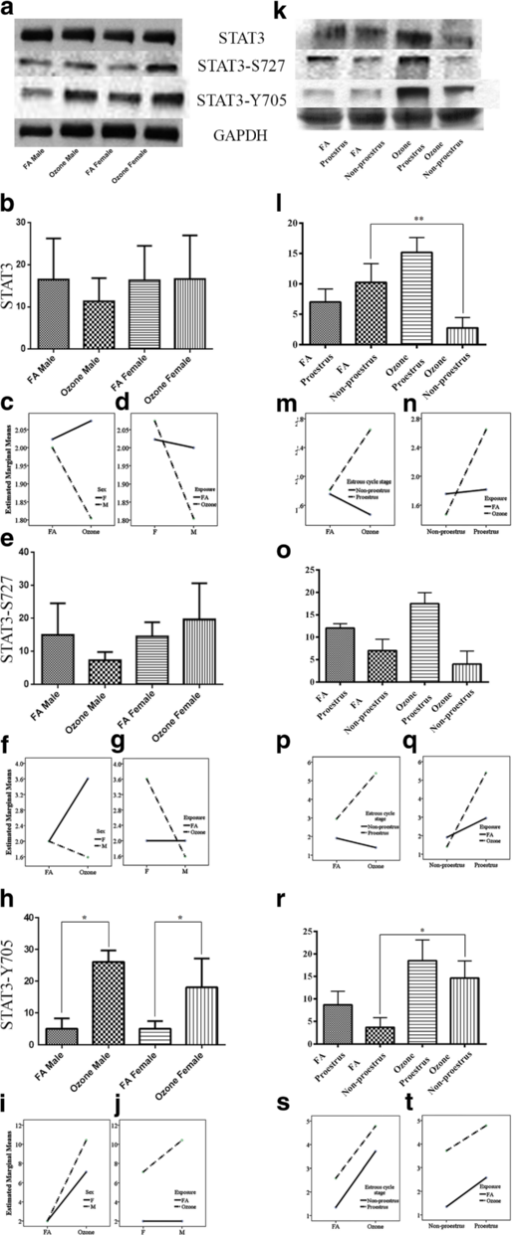
STAT3, STAT3 Serine 727, and STAT3 Tyrosine 705 phosphorylation and effect of ozone exposure. *Left panels*: **a** Representative Western blot images of STAT3, STAT3 Serine 727 and STAT3 Tyrosine 705 phosphorylation expression in males and females with filter air and O_3_ exposure; univariate analysis of STAT3 (**b**), STAT3 Serine 727 (**e**), and STAT3 Tyrosine 705 (**h**), expression in males and females, with FA and O_3_ exposure; Two-way ANOVA interaction effect of sex (**c**) and exposure (**d**), for STAT3 expression, sex (**f**) and exposure (**g**), for STAT3 Serine 727 expression and sex (**i**) and exposure (**j**), for STAT3 Tyrosine 705 expression. *Right panels*: **k** Representative Western blot images of STAT3, STAT3 Serine 727, and STAT3 Tyrosine 705 phosphorylation expression in estrous cycle stages of females, with filter air and O_3_ exposure; univariate analysis of STAT3 (**l**), STAT3 Serine 727 (**o**), and STAT3 Tyrosine 705 (**r**), expression in estrous cycle stages of females, with FA and O_3_ exposure. Two-way ANOVA interaction effect of exposure (**m**) and estrous cycle stages (**n**), for STAT3 expression, exposure (**p**) and estrous cycle stages (**q**), for STAT3 Serine 727 expression, and exposure (**s**) and estrous cycle stages (**t**), for STAT3 Tyrosine 705 expression. Univariate analysis data expressed as Ranks-Kruskal-Wallis test of densitometric analysis; the values are depicted as mean with SD, where *p ≤ 0.05 and **p ≤ 0.01 are the levels of statistical significance compared to controls (*n* = 6–8 per group). Two-way ANOVA for STAT3, STAT3 Serine 727, and STAT3 Tyrosine 705 phosphorylation expression analysis is given in Tables 4, 5, and 6, respectively

One-way analysis of variance revealed no difference in the levels of lung unphosphorylated STAT3 with O_3_ or FA exposure (Additional file 1: Figure S1-c). Similarly, no sex differences were observed in either groups (Fig. 2a, b). Similarly, a two-way ANOVA on the sex and ozone/filter air exposure showed statistically insignificant interaction *F* (1, 52) = 1.765, *p* = 0.190, partial η2 = 0.033. However, analysis of simple main effects for sex and exposure type with Bonferroni adjustment revealed statistically significant but marginal difference in unphosphorylated STAT3 expression score between filter air and ozone exposure. Ozone-exposed females had a very marginal but significant increase in the STAT3 score of 0.135 (95 % CI, 0.003 to 0.267) points compared to the ozone-exposed males, *F* (1, 52) = 4.22, *p* = 0.045, partial η2 = 0.075 (Table 4), whereas males with filter air exposure had an insignificant increase in the STAT3 score of 0.098 (95 % CI, 0.034 to 0.230) points compared to the males exposed to the ozone, *F* (1, 52) = 2.217, *p* = 0.143, partial η2 = 0.041 (Table 4). Interaction effect of sex and exposure for unphosphorylated STAT3 expression is given in Fig. 2c, d.

**Table 4:** Two-way ANOVA for univariate and pairwise comparisons between gender, estrous cycle stages, and exposure for STAT3 expression

Univariate tests: dependent variable: STAT3							
			*df*	*F*	Sig.	Partial η2	
Analysis of gender and exposure	Exposure	FA	1, 52	0.031	0.862	0.001	
		Ozone	1, 52	4.220	0.045	0.075	
	Sex	F	1, 52	0.152	0.698	0.003	
		M	1, 52	2.217	0.143	0.041	
Analysis of female estrous cycle stages and exposure	Estrous cycle stage	Non-proestrus	1, 24	1.916	0.179	0.074	
		Proestrus	1, 24	16.113	0.001	0.402	
	Exposure	FA	1, 24	0.087	0.770	0.004	
		Ozone	1, 24	32.414	< .0005	0.575	
Pairwise comparisons: dependent variable: STAT3							
			(I) Sex	(J) Sex	Mean difference (I–J)	95 % CI for difference	
						Lower bound	Upper bound
Analysis of gender and exposure	Exposure	FA	F	M	0.012	0.120	0.143
		Ozone	F	M	0.135	0.003	0.267
	Sex	F	Ozone	FA	0.026	0.106	0.157
		M	FA	Ozone	0.098	0.034	0.230
Analysis of female estrous cycle stages and exposure	Estrous cycle stage	Non-proestrus	FA	Ozone	0.143	0.070	0.356
		Proestrus	Ozone	FA	0.414	0.201	0.628
	Exposure	FA	Proestrus	Non-proestrus	0.030	0.183	0.244
		Ozone	Proestrus	Non-proestrus	0.588	0.375	0.801

However, one-way analysis of variance of different female estrous cycle stages exerted a significant difference. We found that STAT3 unphosphorylated levels were significantly decreased in females exposed to O_3_ in non-proestrus cycle stages, whereas females exposed in proestrus showed a marked increase with O_3_ exposure compared to the matched control females (Fig. 2k, l). A two-way ANOVA examining the effects of estrous cycle stages and filter air/ozone exposure on the unphosphorylated STAT3 expression showed statistically insignificant interaction *F* (1, 24) = 14.57, *p* = 0.001, partial η2 = 0.378. Analysis of simple main effects for estrous cycle stages and exposure type with Bonferroni adjustment revealed statistically significant difference in unphosphorylated STAT3 expression score. Proestrus females exposed to ozone had a significant increase in the STAT3 score of 0.414 (95 % CI, 0.201 to 0.628) points compared to the filtered air-exposed proestrus females, *F* (1, 24) = 16.113, *p* = 0.001, partial η2 = 0.402 (Table 4). Likewise, ozone-exposed proestrus females had a significant increase in the STAT3 score of 0.588 (95 % CI, 0.375 to 0.801) points compared to the non-proestrus females exposed to ozone, *F* (1, 24) = 32.414, *p* = <0.0005, partial η2 = 0.575 (Table 4). Filter air/ozone exposure on each sex alone and estrous cycle type alone in relation to the unphosphorylated STAT3 is given in Table 4. Interaction effect of exposure and estrous cycle stages for unphosphorylated STAT3 expression is given in Fig. 2m, n.

When we compared he levels of STAT3 S727 phosphorylation in these mice, we found an overall increase with O_3_ exposure (Additional file 1: Figure S1-d). These were comparable in males exposed to O_3_ or FA and in females exposed to FA. However, females exposed to O_3_ displayed a slight but not significant increase in the STAT3 p-S727 levels vs. females exposed to FA (Fig. 2a, e). Two-way ANOVA on the sex and ozone/filter air exposure showed statistically significant interaction *F* (1, 60) = 13.568, *p* = <0.0005, partial η2 = 0.184. Analysis of simple main effects for sex and exposure type with Bonferroni adjustment revealed statistically significant difference in STAT3 S727 phosphorylation expression score between filter air and ozone exposure. Ozone-exposed females had a significant increase in the STAT3 serine 727 score of 1.009 (95 % CI, 0.622 to 1.397) points compared to the ozone-exposed males, *F* (1, 60) = 27.137, *p* = <0.0005, partial η2 = 0.311 (Table 5). Similarly, females with ozone exposure had significant increase in the STAT3 serine 727 score of 0.804 (95 % CI, 0.416 to 1.191) points compared to the females exposed to the filter air, *F* (1, 60) =17.209, *p* = <0.0005, partial η2 = 0.223 (Table 5). Interaction effect of sex and exposure for STAT3 p-S727 expression is given in Fig. 2f, g.

**Table 5:** Two-way ANOVA for univariate and pairwise comparisons between gender, estrous cycle stages, and exposure for STAT3 Serine 727 phosphorylation expression

Univariate tests: dependent variable: STAT3 Serine 727							
			*df*	*F*	Sig.	Partial η2	
Analysis of gender and exposure	Exposure	FA	1, 60	0.001	1.000	0.002	
		Ozone	1,60	27.137	<0.0005	0.311	
	Sex	F	1, 60	17.209	<0.0005	0.223	
		M	1, 60	1.126	0.293	0.018	
Analysis of female estrous cycle stages and exposure	Estrous cycle stage	Non-proestrus	1, 28	2.282	0.142	0.075	
		Proestrus	1, 28	53.117	<0.0005	0.655	
	Exposure	FA	1, 28	9.535	0.005	0.254	
		Ozone	1, 28	141.295	<.0005	0.835	
Pairwise comparisons: dependent variable: STAT3 Serine 727							
			(I) Sex	(J) Sex	Mean difference (I–J)	95 % CI for difference	
						Lower bound	Upper bound
Analysis of gender and exposure	Exposure	FA	F	M	2.500E-8	0.388	0.388
		Ozone	F	M	1.009	0.622	1.397
	Sex	F	Ozone	FA	0.804	0.416	1.191
		M	FA	Ozone	0.206	0.182	0.593
Analysis of female estrous cycle stages and exposure	Estrous cycle stage	Non-proestrus	FA	Ozone	0.254	0.090	0.597
		Proestrus	Ozone	FA	1.223	0.880	1.567
	Exposure	FA	Proestrus	Non-proestrus	0.518	0.174	0.862
		Ozone	Proestrus	Non-proestrus	1.995	1.652	2.339

With one-way analysis of variance, females exposed to O_3_ in proestrus depicted a markedly higher, but not significant, phosphorylation compared to the females exposed in non-proestrus (Fig. 2k, o). Two-way ANOVA examining the effects of estrous cycle stages and filter air/ozone exposure on the STAT3 serine 727 expression showed statistically significant interaction *F* (1, 28) = 38.710, *p* = <0.0005, partial η2 = 0.580. Simple main effects for estrous cycle stages and exposure type with Bonferroni adjustment revealed statistically significant difference in STAT3 serine 727 expression score. Proestrus females exposed to ozone had a significant increase in the STAT3 serine 727 score of 1.223 (95 % CI, 0.880 to 1.567) points compared to the filtered air-exposed proestrus females, *F* (1, 28) = 53.117, *p* = <0.0005, partial η2 = 0.655 (Table 5). Similarly, ozone-exposed proestrus females had a significant increase in the STAT3 serine 727 score of 1.995 (95 % CI, 1.652 to 2.339) points compared to the non-proestrus females exposed to ozone, *F* (1, 28) = 141.295, *p* = <0.0005, partial η2 = 0.835 (Table 5). Filter air/ozone exposure on each sex alone and estrous cycle type alone in relation to the unphosphorylated STAT3 serine 727 phosphorylation is given in Table 5. Interaction effect of exposure and estrous cycle stages for SATAT3 p-S727 expression is given in Fig. 2p, q.

Univariate analysis of lung STAT3 Y705 phosphorylation revealed a significant overall increase in animals exposed to O_3_ compared to FA (Additional file 1: Figure S1-e) irrespective of sex differences (Fig. 2a, h). Two-way ANOVA on the sex and ozone/filter air exposure also showed statistically significant interaction *F* (1, 52) = 12.40, *p* = 0.001, partial η2 = 0.193. Analysis of simple main effects for sex and exposure type with Bonferroni adjustment revealed statistically significant difference in STAT3 tyrosine 705 phosphorylation expression score between filter air and ozone exposure. Ozone-exposed male had significant increase in the STAT3 tyrosine 705 score of 1.645 (95 % CI, 0.982 to 2.308) points compared to the ozone-exposed females, *F* (1, 52) = 24.80, *p* = <0.0005, partial η2 = 0.323 (Table 6) whereas males with ozone exposure had insignificant increase in the STAT3 tyrosine 705 score of 4.214 (95 % CI, 3.35 to 4.87) points compared to the males exposed to the filter air, *F* (1, 52) = 162.686, *p* = <0.0005, partial η2 = 0.758 (Table 6). Interaction effect of sex and exposure for STAT3 p-Y705 expression is given in Fig. 2i, j.

**Table 6:** Two-way ANOVA for univariate and pairwise comparisons between gender, estrous cycle stages, and exposure for STAT3 Tyrosine 705 phosphorylation expression

Univariate tests: dependent variable: STAT3 Tyrosine 705							
			*df*	*F*	Sig.	Partial η2	
Analysis of gender and exposure	Exposure	FA	1, 52	0.001	1.000	0.001	
		Ozone	1, 52	24.800	<0.0005	0.323	
	Sex	F	1, 52	60.448	<0.0005	0.538	
		M	1, 52	162.686	<0.0005	0.758	
Analysis of female estrous cycle stages and exposure	Estrous cycle stage	Non-proestrus	1, 28	58.045	<0.0005	0.675	
		Proestrus	1, 28	50.192	<0.0005	0.642	
	Exposure	FA	1, 28	15.759	<0.0005	0.360	
		Ozone	1, 28	11.804	0.002	0.297	
Pairwise comparisons: dependent variable: STAT3 Tyrosine 705							
			(I) Sex	(J) Sex	Mean difference (I–J)	95 % CI for difference	
						Lower bound	Upper bound
Analysis of gender and exposure	Exposure	FA	M	F	1.429E−8	0.663	0.663
		Ozone	M	F	1.645	0.982	2.308
	Sex	F	Ozone	FA	2.569	1.906	3.232
		M	Ozone	FA	4.214	3.551	4.877
Analysis of female estrous cycle stages and exposure	Estrous cycle stage	Non-proestrus	Ozone	FA	1.185	0.866	1.504
		Proestrus	Ozone	FA	1.102	0.783	1.421
	Exposure	FA	Proestrus	Non-proestrus	0.617	0.299	0.936
		Ozone	Proestrus	Non-proestrus	0.534	0.216	0.853

Ozone exposure in females in proestrus exerted a slightly higher expression change in the STAT3 p-Y705 compared to females exposed in non-proestrus stages, but unilabiate analysis exhibited no significant differences between these groups (Fig. 2k, r). Similarly, two-way ANOVA examining the effects of estrous cycle stages and filter air/ozone exposure on the STAT3 tyrosine 705 expression showed statistically insignificant interaction *F* (1, 28) = 0.143, *p* = 0.709, partial η2 = 0.005. However, simple main effects for estrous cycle stages and exposure type with Bonferroni adjustment revealed statistically significant difference in STAT3 tyrosine 705 expression score. Non-proestrus female exposed to ozone had significant increase in the STAT3 tyrosine 705 score of 1.185 (95 % CI, 0.866 to 1.504) points compared to the filtered air-exposed non-proestrus female, *F* (1, 28) = 58.045, *p* = <0.0005, partial η2 = 0.675 (Table 6) whereas filtered air-exposed proestrus females had significant increase in the STAT3 tyrosine 705 score of 0.617 (95 % CI, 0.299 to 0.936) points compared to the non-proestrus females exposed to the filter air, *F* (1, 28) =15.759, *p* = <0.0005, partial η2 = 0.360 (Table 6). Filter air/ozone exposure on each sex alone and estrous cycle type alone in relation to the unphosphorylated STAT3 tyrosine 705 phosphorylation is given in Table 6. Interaction effect of exposure and estrous cycle stages for STAT3 p-Y705 expression is given in Fig. 2s, t.

### Ozone-associated lung inflammation and expression of JAK2 and JAK2 phosphorylation

Interleukin-6 preferentially activates STAT3 with phosphorylation of Y705 via the JAK signaling pathway. To assess activation of this mechanism in our model, we measured the levels of JAK2 and JAK2 phosphorylation (Y1007+Y1008) with relation to O_3_ and FA exposure and further comparison of sex differences and female estrous cycle.

**Fig. 3:**
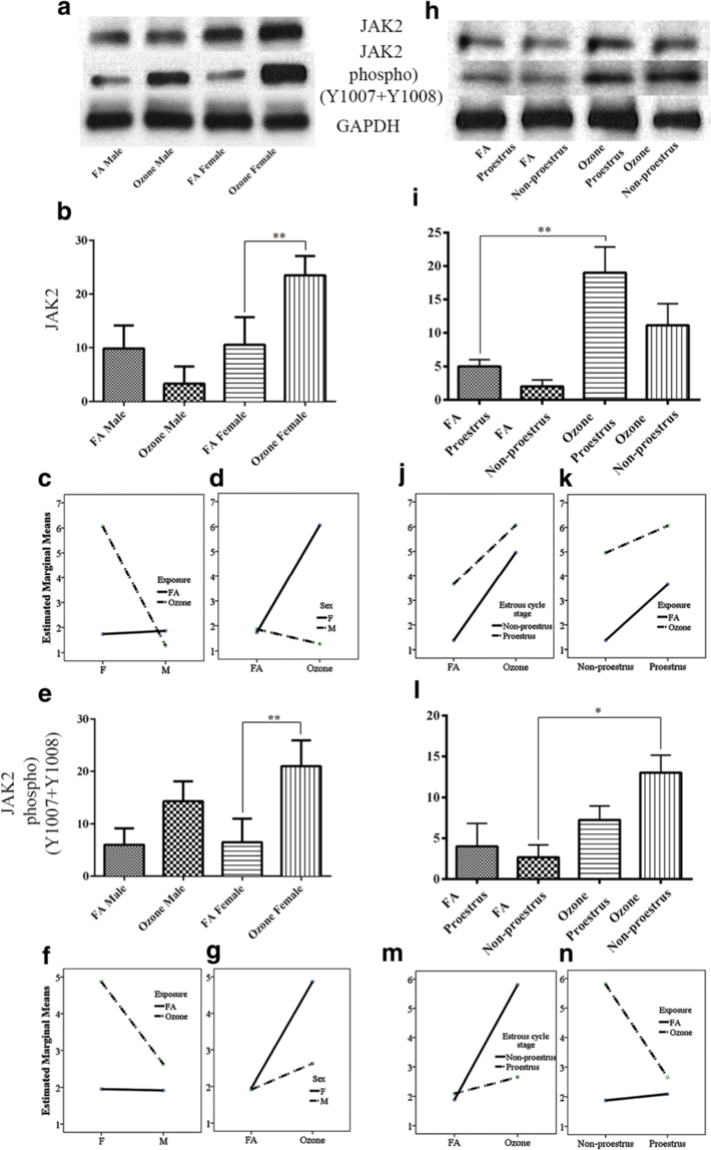
JAK2 and JAK2 phosphorylated (Y1007+Y1008) expression and effect of ozone exposure. *Left panels*: **a** Representative Western blot images of JAK2 and JAK2 phosphorylated (Y1007+Y1008) expression in males and females with filter air and O_3_ exposure; univariate analysis of JAK2 (**b**) and JAK2 (**e**) phosphorylated (Y1007+Y1008), expression in males and females, with FA and O_3_ exposure; two-way ANOVA interaction effect of sex (**c**) and exposure (**d**), for JAK2 expression and sex (**f**) and exposure (**g**), for JAK2 phosphorylated (Y1007+Y1008) expression. *Right panels*: **h** Representative Western blot images of JAK2 and JAK2 phosphorylated (Y1007+Y1008) expression in estrous cycle stages of females, with filter air and O_3_ exposure; univariate analysis of **i** JAK2 and **l** JAK2 phosphorylated (Y1007+Y1008), expression in estrous cycle stages of females, with FA and O_3_ exposure. Two-way ANOVA interaction effect of exposure (**j**) and estrous cycle stages (**k**), for JAK2 expression and exposure (**m**) and estrous cycle stages (**n**) and for JAK2 phosphorylated (Y1007+Y1008) expression. Univariate analysis data expressed as Ranks-Kruskal-Wallis test of densitometric analysis; the values are depicted as mean with SD, where *p ≤ 0.05 and **p ≤ 0.01 are the levels of statistical significance compared to controls (*n* = 6–8 per group). Two-way ANOVA for JAK2 and JAK2 phosphorylated (Y1007+Y1008) analysis is given in Tables 7 and 8, respectively

Irrespective of sex differences, one-way analysis of variance of pooled data showed a significant increase in the expression of JAK2 unphosphorylated with O_3_ exposure compared to animals exposed to FA (Additional file 1: Figure S1-f). However, comparison of males and females represented deviations in the expression patterns, where O_3_ exposure resulted in an overall decrease in JAK2 expression in males, while females had a significant increase in JAK2 expression (Fig. 3a, b). Two-way ANOVA on the sex and ozone/filter air exposure showed statistically significant interaction *F* (1, 52) = 627.9, *p* = <0.0005, partial η2 = 0.924. Analysis of simple main effects for sex and exposure type with Bonferroni adjustment revealed statistically significant difference in unphosphorylated JAK2 expression score between filter air and ozone exposure. Ozone-exposed female had significant increase in the unphosphorylated JAK2 expression score of 2.395 (95 % CI, 2.225 to 2.543) points compared to the ozone-exposed males, *F* (1, 52) = 1190.013, *p* = <0.0005, partial η2 = 0.958 (Table 7) whereas females with ozone exposure had significant increase in the unphosphorylated JAK2 score of 2.165 (95 % CI, 2.026 to 2.304) points compared to the females exposed to the filter air, *F* (1, 52) =972.74, *p* = <0.0005, partial η2 = 0.942 (Table 7). Interaction effect of sex and exposure for JAK2 expression is given in Fig. 3c, d.

**Table 7:** Two-way ANOVA for univariate and pairwise comparisons between gender, estrous cycle stages, and exposure for JAK2 expression

Univariate tests: dependent variable: JAK2							
			*df*	*F*	Sig.	Partial η2	
Analysis of gender and exposure	Exposure	FA	1, 52	0.890	0.350	0.017	
		Ozone	1, 52	1190.01	<0.0005	0.958	
	Sex	F	1, 52	972.745	<0.0005	0.949	
		M	1, 52	18.071	<0.0005	0.258	
Analysis of female estrous cycle stages and exposure	Estrous cycle stage	Non-proestrus	1, 36	360.191	<0.0005	0.909	
		Proestrus	1, 36	161.757	<0.0005	0.818	
	Exposure	FA	1, 36	147.012	<0.0005	0.803	
		Ozone	1, 36	34.392	<0.0005	0.489	
Pairwise comparisons: dependent variable: JAK2							
			(I) Sex	(J) Sex	Mean difference (I–J)	95 % CI for difference	
						Lower bound	Upper bound
Analysis of gender and exposure	Exposure	FA	M	F	0.065	0.074	0.205
		Ozone	F	M	2.395	2.255	2.534
	Sex	F	Ozone	FA	2.165	2.026	2.304
		M	FA	Ozone	0.295	0.156	0.434
Analysis of female estrous cycle stages and exposure	Estrous cycle stage	Non-proestrus	Ozone	FA	1.795	1.603	1.987
		Proestrus	Ozone	FA	1.203	1.011	1.395
	Exposure	FA	Proestrus	Non-proestrus	1.147	0.955	1.339
		Ozone	Proestrus	Non-proestrus	0.555	0.363	0.747

Univariate analysis of females at different estrous cycle stages revealed a higher and significant increase in the levels of unphosphorylated JAK2 in females exposed to O_3_ in proestrus compared to females exposed in non-proestrus stages and females exposed to FA (Fig. 3h, i). Two-way ANOVA examining the effects of estrous cycle stages and filter air/ozone exposure showed statistically significant interaction *F* (1, 36) = 19.596, *p* = <0.0005, partial η2 = 0.352. Simple main effects for estrous cycle stages and exposure type with Bonferroni adjustment revealed statistically significant difference in unphosphorylated JAK2 expression score. Ozone-exposed non-proestrus females had significant increase in the unphosphorylated JAK2 score of 1.795 (95 % CI, 1.603 to 1.987) points compared to the non-proestrus females exposed to the filter air, *F* (1, 36) = 360.191, *p* = <0.0005, partial η2 = 0.909 (Table 7). Similarly, proestrus female exposed to ozone had significant increase in the unphosphorylated JAK2 score of 0.555 (95 % CI, 0.363 to 0.747) points compared to the ozone-exposed non-proestrus female, *F* (1, 36) = 34.392, *p* = <0.0005, partial η2 = 0.489 (Table 7). Filter air/ozone exposure on each sex alone and estrous cycle type alone in relation to the unphosphorylated JAK2 is given in Table 7. Interaction effect of exposure and estrous cycle stages for JAK2 expression is given in Fig. 3j, k.

JAK2 phosphorylation (Y1007+Y1008) showed an overall significant increase with O_3_ exposure compared to FA (Additional file 1: Figure S1g). Females showed a higher and significant expression of phosphorylated JAK2 (Y1007+Y1008) compared to O_3_-exposed males (Fig. 3a, e). Two-way ANOVA on the sex and ozone/filter air exposure showed statistically significant interaction *F* (1, 52) = 23.991, *p* = <0.0005, partial η2 = 0.316. Analysis of simple main effects for sex and exposure type with Bonferroni adjustment revealed statistically significant difference in phosphorylated JAK2 expression score between filter air and ozone exposure. Ozone-exposed female had significant increase in the phosphorylated JAK2 expression score of 1.122 (95 % CI, 0.779 to 1.465) points compared to the ozone-exposed males, *F* (1, 52) = 43.108, *p* = <0.0005, partial η2 = 0.453 (Table 8) whereas females with ozone exposure had significant increase in the phosphorylated JAK2 score of 1.538 (95 % CI, 1.195 to 1.881) points compared to the females exposed to the filter air, *F* (1, 52) = 80.967, *p* = <0.0005, partial η2 = 0.609 (Table 8). Interaction effect of sex and exposure for JAK2 p-(Y1007+Y1008) expression is given in Fig. 3f, g.

**Table 8:** Two-way ANOVA for univariate and pairwise comparisons between gender, estrous cycle stages, and exposure for JAK2 phosphorylation (Y1007+Y1008) expression

Univariate tests: dependent variable: JAK2 phosphorylated (Y1007+Y1008)							
			*df*	*F*	Sig.	Partial η2	
Analysis of gender and exposure	Exposure	FA	1, 52	0.131	0.719	0.003	
		Ozone	1, 52	43.108	<0.0005	0.453	
	Sex	F	1, 52	80.967	<0.0005	0.609	
		M	1, 52	4.290	0.043	0.076	
Analysis of female estrous cycle stages and exposure	Estrous cycle stage	Non-proestrus	1, 24	253.484	<0.0005	0.914	
		Proestrus	1, 24	5.101	0.033	0.175	
	Exposure	FA	1, 24	0.764	0.391	0.031	
		Ozone	1, 24	163.543	<0.0005	0.872	
Pairwise comparisons: dependent variable: JAK2 phosphorylated (Y1007+Y1008)							
			(I) Sex	(J) Sex	Mean difference (I–J)	95 % CI for difference	
						Lower bound	Upper bound
Analysis of gender and exposure	Exposure	FA	M	F	0.062	0.281	0.405
		Ozone	F	M	1.122	0.779	1.465
	Sex	F	Ozone	FA	1.538	1.195	1.881
		M	Ozone	FA	0.354	0.011	0.697
Analysis of female estrous cycle stages and exposure	Estrous cycle stage	Non-proestrus	Ozone	FA	1.965	1.710	2.220
		Proestrus	Ozone	FA	0.279	0.024	0.534
	Exposure	FA	Proestrus	Non-proestrus	0.108	0.147	0.363
		Ozone	Non-proestrus	Proestrus	1.578	1.324	1.833

However, as opposed to unphosphorylated JAK2, phosphorylated JAK2 (Y1007+Y1008) was found to have a higher and significant increase in females exposed to O_3_ in non-proestrus stages vs. females exposed in proestrus (Fig. 3h, l). Two-way ANOVA examining the effects of estrous cycle stages and filter air/ozone exposure showed statistically significant interaction *F* (1, 24) = 93.334, *p* = <0.0005, partial η2 = 0.795. Simple main effects for estrous cycle stages and exposure type with Bonferroni adjustment revealed statistically significant difference in phosphorylated JAK2 expression score. Ozone-exposed non-proestrus females had significant increase in the phosphorylated JAK2 score of 1.965 (95 % CI, 1.710 to 2.220) points compared to the non-proestrus females exposed to the filter air, *F* (1, 24) = 253.484, *p* = <0.0005, partial η2 = 0.914 (Table 8). Similarly, non-proestrus female exposed to ozone had significant increase in the phosphorylated JAK2 score of 1.578 (95 % CI, 1.324 to 1.883) points compared to the ozone-exposed proestrus female, *F* (1, 24) = 163.543, *p* = <0.0005, partial η2 = 0.872 (Table 8). Filter air/ozone exposure on each sex alone and estrous cycle type alone in relation to the phosphorylated JAK2 is given in Table 8. Interaction effect of exposure and estrous cycle stages for JAK2 p-(Y1007+Y1008) expression is given in Fig. 3m, n.

### Ozone-associated lung inflammation and expression of NF-κB (p105/p50)

**Fig. 4:**
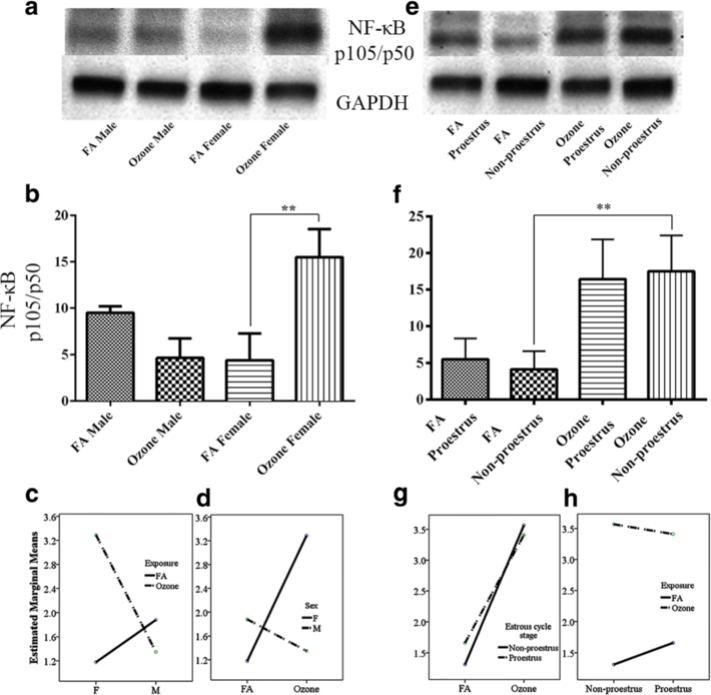
NF-κB (p105/p50) expression and effect of ozone exposure. *Left panel*: **a** Representative Western blot images of NF-κB (p105/p50) expression in males and females with filter air and O_3_ exposure; **b** univariate analysis of NF-κB (p105/p50) expression in males and females with FA and O_3_ exposure; two-way ANOVA interaction effect of sex (**c**) and exposure (**d**) for NF-κB (p105/p50) expression. *Right panel*: **e** Representative Western blot images of NF-κB (p105/p50) expression in estrous cycle stages of females, with filter air and O_3_ exposure; **f** univariate analysis of NF-κB (p105/p50) expression in estrous cycle stages of females with FA and O_3_ exposure. Two-way ANOVA interaction effect of exposure (**g**) and estrous cycle stages (**h**) for NF-κB (p105/p50) expression. Univariate analysis data expressed as Ranks-Kruskal-Wallis test of densitometric analysis; the values are depicted as mean with SD, where **p ≤ 0.01 is the level of statistical significance compared to controls (*n* = 6–8 per group). Two-way ANOVA for NF-κB (p105/p50) expression analysis is given in Table 9

NF-κB activation is widely implicated in inflammatory conditions and is also known to possess cross-talk with pathways that may influence IL-6 expression. IL-6 via STAT3 transcription effector mediates local vascular macrophage activation in lungs and protection from oxidative stress. In addition, the NF-κB–IL-6 signaling pathway plays multiple roles in initiating and sustaining vascular inflammation. Assessment of NF-κB expression in our model showed sex differences in the lungs of mice exposed to O_3_ or FA. Ozone-exposed male mice showed a reduction in NF-κB expression, whereas females exposed to O_3_ had a significant increase in the NF-κB expression (Fig. 4a, b). However, due to sheer increase in the expression of NF-κB in females, pooling male and female data together masked the decrease in the expression in males, and an overall increase in expression was found with O_3_ exposure (Additional file 1: Figure S1-h). Two-way ANOVA on the sex and ozone/filter air exposure showed statistically significant interaction *F* (1, 52) = 266.435, *p* = <0.0005, partial η2 = 0.837. Analysis of simple main effects for sex and exposure type with Bonferroni adjustment revealed statistically significant difference in NF-κB (p105/p50) expression score between filter air and ozone exposure. Ozone-exposed female had significant increase in the NF-κB (p105/p50) expression score of 0.970 (95 % CI, 0.855 to 1.085) points compared to the ozone-exposed males, *F* (1, 52) = 287.977, *p* = <0.0005, partial η2 = 0.847 (Table 9), whereas females with ozone exposure had significant increase in the NF-κB (p105/p50) score of 1.056 (95 % CI, 0.941 to 1.170) points compared to the females exposed to the filter air, *F* (1, 52) = 341.072, *p* = <0.0005, partial η2 = 0.868 (Table 9). Interaction effect of sex and exposure for NF-κB expression is given in Fig. 4c, d.

**Table 9:** Two-way ANOVA for univariate and pairwise comparisons between gender, estrous cycle stages, and exposure for NF-κB (p105/p50) expression

Univariate tests: dependent variable: NF-κB (p105/p50)							
			*df*	*F*	Sig.	Partial η2	
Analysis of gender and exposure	Exposure	FA	1,52	37.382	<0.0005	0.418	
		Ozone	1,52	287.977	<0.0005	0.847	
	Sex	F	1,52	341.072	<0.0005	0.868	
		M	1,52	21.306	<0.0005	0.291	
Analysis of female estrous cycle stages and exposure	Estrous cycle stage	Non-proestrus	1,32	148.459	<0.0005	0.823	
		Proestrus	1,32	89.059	<0.0005	0.736	
	Exposure	FA	1,32	3.581	0.068	0.101	
		Ozone	1,32	0.731	0.399	0.022	
Pairwise comparisons: dependent variable: NF-κB (p105/p50)							
			(I) Sex	(J) Sex	Mean difference (I–J)	95 % CI for difference	
						Lower bound	Upper bound
Analysis of gender and exposure	Exposure	FA	M	F	0.349	0.235	0.464
		Ozone	F	M	0.970	0.855	1.085
	Sex	F	Ozone	FA	1.056	0.941	1.170
		M	FA	Ozone	0.264	0.149	0.379
Analysis of female estrous cycle stages and exposure	Estrous cycle stage	Non-proestrus	Ozone	FA	1.129	0.940	1.318
		Proestrus	Ozone	FA	0.874	0.686	1.063
	Exposure	FA	Proestrus	Non-proestrus	0.175	0.013	0.364
		Ozone	Non-proestrus	Proestrus	0.079	.110	0.268

Univariate analysis of females exposed in different estrous cycle stages showed no overall difference in the expression patterns of the NF-κB with O_3_ exposure, compared to the matched controls exposed to the FA (Fig. 4e, f). Examining the effects of estrous cycle stages and filter air/ozone exposure through two-way ANOVA also showed statistically insignificant interaction *F* (1, 32) = 3.774, *p* = <0.061, partial η2 = 0.105. Simple main effects for estrous cycle stages and exposure type with Bonferroni adjustment revealed statistically significant difference in NF-κB expression score. Ozone-exposed non-proestrus females had significant increase in the NF-κB score of 1.129 (95 % CI, 0.940 to 1.318) points compared to the non-proestrus females exposed to the filter air, *F* (1, 32) = 148.459, *p* = <0.0005, partial η2 = 0.823 (Table 9). Similarly, proestrus female exposed to filter had insignificant increase in the NF-κB score of 0.175 (95 % CI, 0.013 to 0.363) points compared to the filtered air-exposed non-proestrus female, *F* (1, 32) = 3.581, *p* = 0.068, partial η2 = 0.101 (Table 9). Filter air/ozone exposure on each sex alone and estrous cycle type alone in relation to the NF-κB is given in Table 9. Interaction effect of exposure and estrous cycle stages for NF-κB expression is given in Fig. 4g, h.

### Ozone-associated lung inflammation and expression of AKT1

**Fig. 5:**
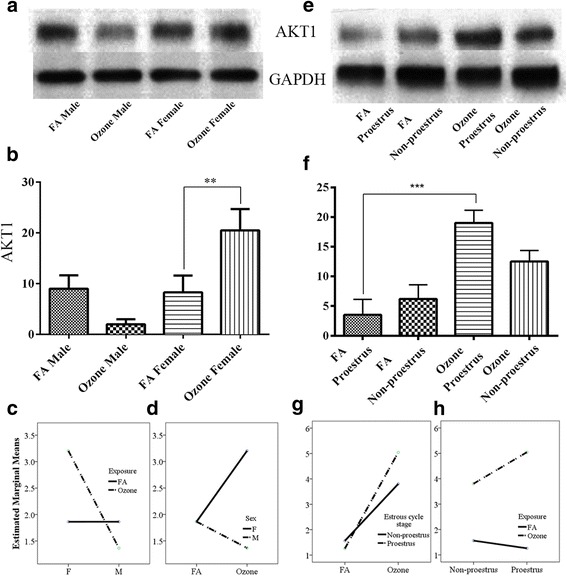
AKT1 expression and effect of ozone exposure. *Left panel*: **a** Representative Western blot images of AKT1 expression in males and females with filter air and O_3_ exposure; **b** univariate analysis of AKT1 expression in males and females with FA and O_3_ exposure; two-way ANOVA interaction effect of sex (**c**) and exposure (**d**) for AKT1 expression. *Right panels*: **e** Representative Western blot images of AKT1 expression in estrous cycle stages of females, with filter air and O_3_ exposure; **f** univariate analysis of AKT1 expression in estrous cycle stages of females with FA and O_3_ exposure. Two-way ANOVA interaction effect of exposure (**g**) and estrous cycle stages (**h**) for AKT1 expression. Univariate analysis data expressed as Ranks-Kruskal-Wallis test of densitometric analysis; the values are depicted as mean with SD where **p ≤ 0.01 and ***p ≤ 0.001 are the levels of statistical significance compared to controls (*n* = 6–8 per group). Two-way ANOVA for AKT1 expression analysis is given in Table 10

Studies have documented that IL-6/STAT3 signaling can regulate AKT1 activation and that both JAK2 and AKT1 may play role in the activation of NF-κB [48, 49]. Initially believed to operate as components of distinct signaling pathways, several studies have demonstrated that the NF-κB and AKT1 signaling pathways can converge and play a crucial role in stress responses and inflammation [50]. In our experimental model, ozone exposure in pooled male and female mice resulted in no differences in AKT1 expression compared to animals exposed to FA (Additional file 1: Figure S1i). However, analysis of sex differences in lung AKT1 levels in response to O_3_ exposure depicted a marked decrease in males compared to matched controls exposed to FA, while females exposed to O_3_ showed a significant increase vs. FA (Fig. 5a, b).

Two-way ANOVA on the sex and ozone/filter air exposure showed statistically significant interaction *F* (1, 52) = 60.730, *p* = <0.0005, partial η2 = 0.539. Analysis of simple main effects for sex and exposure type with Bonferroni adjustment revealed statistically significant difference in AKT1 expression score between filter air and ozone exposure. Ozone-exposed female had significant increase in the AKT1 expression score of 0.919 (95 % CI, 0.751 to 1.086) points compared to the ozone-exposed males, *F* (1, 52) = 121.354, *p* = <0.0005, partial η2 = 0.70 (Table 10) whereas females with ozone exposure had significant increase in the AKT1 score of 0.670 (95 % CI, 0.503 to 0.837) points compared to the females exposed to the filter air, *F* (1, 52) = 64.526, *p* = <0.0005, partial η2 = 0.554 (Table 10). Interaction effect of sex and exposure for AKT1 expression is given in Fig. 5c, d.

**Table 10:** Two-way ANOVA for univariate and pairwise comparisons between gender, estrous cycle stages and exposure for AKT1 expression

Univariate tests: dependent variable: AKT1							
			*df*	*F*	Sig.	Partial η2	
Analysis of gender and exposure	Exposure	FA	1,52	0.001	0.996	0.002	
		Ozone	1,52	121.354	<0.0005	0.700	
	Sex	F	1,52	64.526	<0.0005	0.554	
		M	1,52	8.929	0.004	0.147	
Analysis of female estrous cycle stages and exposure	Estrous cycle stage	Non-proestrus	1,24	179.278	<0.0005	0.882	
		Proestrus	1,24	511.045	<0.0005	0.955	
	Exposure	FA	1,24	3.261	0.084	0.120	
		Ozone	1,24	54.923	<0.0005	0.696	
Pairwise comparisons: dependent variable: AKT1							
			(I) Sex	(J) Sex	Mean difference (I–J)	95 % CI for difference	
						Lower bound	Upper bound
Analysis of gender and exposure	Exposure	FA	F	M	0.001	0.167	0.168
		Ozone	F	M	0.919	0.751	1.086
	Sex	F	Ozone	FA	0.670	0.503	0.837
		M	FA	Ozone	0.249	0.082	0.417
Analysis of female estrous cycle stages and exposure	Estrous cycle stage	Non-proestrus	Ozone	FA	1.121	0.948	1.293
		Proestrus	Ozone	FA	1.892	1.719	2.065
	Exposure	FA	Non-proestrus	Proestrus	0.151	0.022	0.324
		Ozone	Proestrus	Non-proestrus	0.620	0.448	0.793

Examination of lung AKT1 levels in females exposed to O_3_ at different stages of the estrous cycle showed an increased expression in animals exposed in both proestrus and non-proestrus, but only females exposed in proestrus had a significant increase in AKT1 (Fig. 5e, f). The effects of estrous cycle stages and filter air/ozone exposure through two-way ANOVA also showed statistically significant interaction *F* (1, 24) = 42.745, *p* = < 0.0005, partial η2 = 0.639. Simple main effects for estrous cycle stages and exposure type with Bonferroni adjustment revealed statistically significant difference in AKT1 expression score. Ozone-exposed proestrus females had significant increase in the AKT1 score of 1.892 (95 % CI, 1.719 to 2.065) points compared to the proestrus females exposed to the filter air, *F* (1, 24) = 511.045, *p* = <0.0005, partial η2 = 0.955 (Table 10). Similarly, proestrus female exposed to ozone had significant increase in the AKT1 score of 0.620 (95 % CI, 0.448 to 0.793) points compared to the ozone-exposed non-proestrus female, *F* (1, 24) = 54.923, *p* = <0.0005, partial η2 = 0.696 (Table 10). Filter air/ozone exposure on each sex alone and estrous cycle type alone in relation to the AKT1 is given in Table 10. Interaction effect of exposure and estrous cycle stages for AKT1 expression is given in Fig. 5g, h.

## Discussion

Innate immunity plays a critical role against infection and oxidative damage from inhaled air pollutants. Acute airway responses to inhaled ground-level O_3_ are characterized by recruitment of inflammatory cells to the lung epithelium and by the generation of inflammatory mediators including cytokines, chemokines, and adhesion molecules [51]. The specific mechanisms of O_3_ toxicity appear to be related to oxidation of cell membranes and surfactant, resulting in lipid peroxidation and the production of reactive oxygen species [52]. The resulting oxidation products can prime alveolar macrophages and induce an increase in the production of pro-inflammatory cytokines that can result in injury of the lung epithelium, affecting its normal function and the overall lung innate immunity. Although studies have reported differential outcomes for lung disease triggered by ambient air pollution in men and women, the associated mechanisms of the immune response to O_3_ in the male and female lung remain unknown [53–55].

It has been known for years that the clinical course of inflammatory lung disease is highly influenced by sex, hormones, and the environment [56, 57]. In our previous work, we demonstrated that expression of inflammatory mediators varies with sex in response to acute O_3_ exposure, indicating that fluctuating sex hormones levels may affect the immune response to environmental challenges [39]. Specifically, we reported differential mRNA expression levels of immune-related genes including pattern recognition receptors, transcription factors, and immune response mediators in the lungs of male and female mice exposed to O_3_ or FA, with significantly altered expression levels of neutrophil-attracting chemokines, oxidative stress-related enzymes, and pro-inflammatory cytokines such as IL-6 [39]. Studies have indicated a complex and conflicting role for IL-6 in the lung injury. Overexpression of IL-6 greatly reduces hyperoxic lung injury in the transgenic mice [58]. Similarly, in an aerosolized endotoxin model of lung injury, endogenous IL-6 was found to be associated with decreased levels of TNF-α, MIP-2, GM-CSF, IFNgamma, and airspace neutrophils and thereby exhibits a crucial anti-inflammatory function. On the contrary, various other clinical and experimental studies establish deleterious function of IL-6 in acute lung injury. Clinical and animal studies both have suggested a role of IL-6 in the pathogenesis of ventilator-associated lung injury [59–62]. Similarly, in a mouse sepsis model, IL-6 was found to be associated with increased mortality and increased lung complement 5a receptor expression [63]. IL-6 plays an important and essential role in activating STAT transcription factors and thereby increases the recruitment of neutrophils in lung. In our animal model, we have previously demonstrated sex differences in the lung vascular permeability and polymorphonuclear neutrophil content in response to ozone exposure [39]. Marked increase in the neutrophil-attracting chemokines (Ccl20, Cxcl5, and Cxcl2) and pro-inflammatory cytokine IL-6 mRNAs with ozone exposure along with sex differences warrants further studies to examine the IL-6 and its sequential downstream pathways for mechanism of ozone-associated lung damage and higher susceptibility and severity of lung diseases in females. Better understating of the mechanism of the ozone-induced lung damage and sex differences will pave path for the research and discovery of therapeutic targets for patients with acute lung injury.

In this work, we have further characterized the acute lung immune response to O_3_ in the male and female lung at the intracellular level, by identifying sex-specific intracellular signaling associated with IL-6. We also evaluated a potential contribution of the estrous cycle to this regulation, in order to investigate whether circulating female hormones contribute to O_3_-induced inflammatory responses by modulating lung gene expression changes. Our results showed sex differences in protein expression and phosphorylation of various components of the IL-6/IL-6R intracellular pathway and suggested a correlation of some of these with increases in pre-ovulatory hormone levels in the afternoon of proestrus. Because the lung expresses both estrogen and progesterone receptors, and these control alveolar loss and regeneration processes [64, 65], it is likely that ovarian hormones play a central role in lung inflammation and injury by modulating of immune gene expression [66]. In this regard, future studies using models of gonadectomy and hormone replacement will likely unveil the specific roles of sex hormones in the inflammatory response to O_3_.

**Fig. 6:**
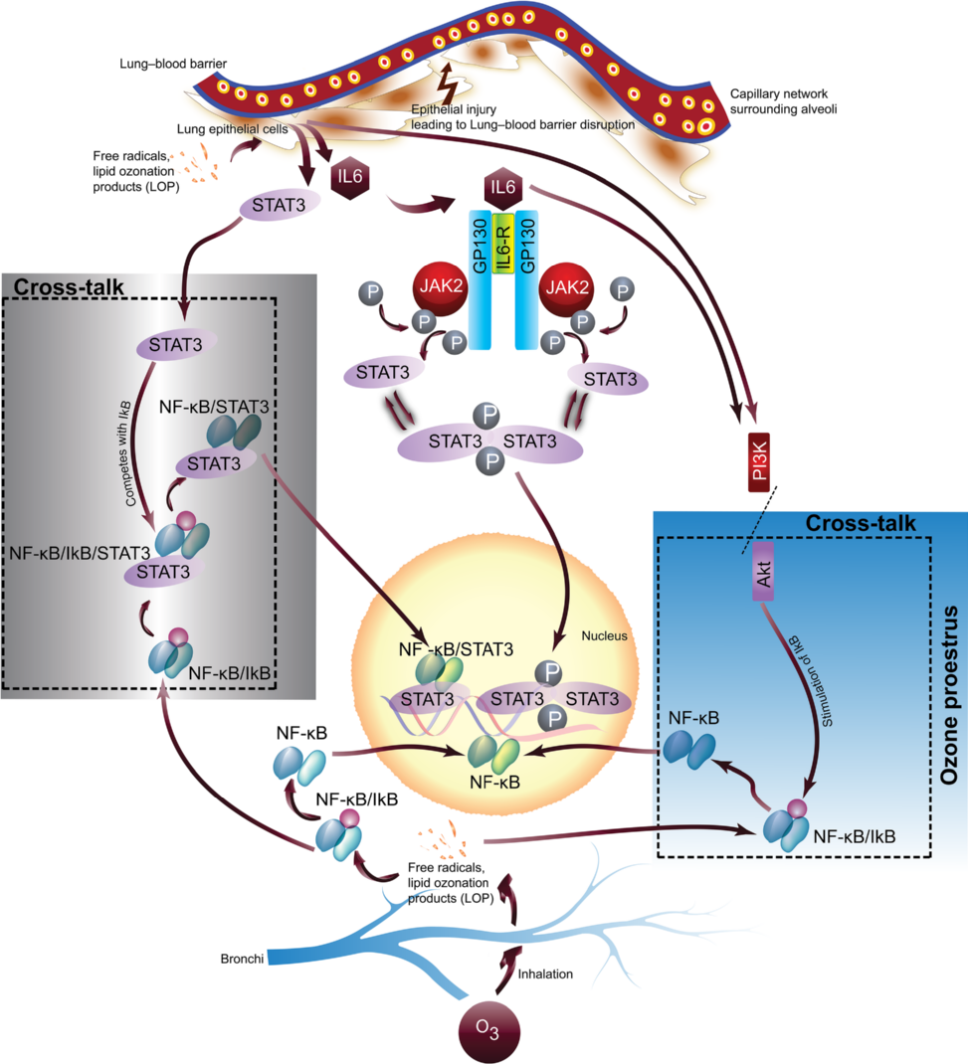
Schematic of ozone-induced lung inflammation and role of IL-6 signaling pathway: ozone-associated lung inflammation is primarily driven by lipid ozonation products (LOP) and generation of free radicals primarily in lung epithelial cells. LOP and free radicals can further mediate activation of downstream biochemical events and cascades of secondary cellular responses leading to the inflammatory damage. The production of free radicals and LOP can trigger (i) increased expression of IL-6 and STAT3 and (ii) activation of NF-κB and (iii) activation of PI3K/AKT pathway

Interleukin-6 is secreted by immune cells and lung endothelial and epithelial cells in response to environmental insults [67, 68]. This cytokine is known for its pleiotropic effects in mediating the pathogenesis, progression, and severity of various chronic lung diseases [69–71]. Binding of IL-6 to its receptor activates the signal transducing receptor glycoprotein 130 (gp130), inducing homodimerization and activation of Janus kinases (JAKs) that in turn activate signal transducers and activators of transcription (AKT1, NF-κB, STAT3) [72–74]. Phosphorylation of STAT3 allows for dimerization and nuclear translocation, where it can bind to specific elements in gene promoters and regulate their expression [75]. Our results strongly advocate for the co-existence of canonical and non-canonical STAT3 signaling mechanisms in the O_3_ induced oxidative lung damage (Fig. 6). A very minor alteration in the level of non-phosphorylated STAT3 in O_3_ exposed males and lowering of the non-phosphorylated STAT3 in O_3_ exposed non-proestrus females is a suggestive of canonical mechanism of STAT3 nuclear translocation. Conversely, up-regulation of non-phosphorylated STAT3 in O_3_ exposed females in proestrus arguments for the non-canonical mechanism. Based on our results, we hypothesized that dual converging pathways which are acting synergistically in response to the level and type of hormones presented during the O_3_ insult dictate the inflammatory response and severity of the lung damage (Fig. 6). Our data suggest that O_3_ induced oxidative stress acts as a dual-edged sword for the dysfunction of the lung alveolar epithelial barrier and alveolar-capillary endothelial barrier. The oxidative damage by itself compromise the barrier function integrity and also in turn promote expression of increased levels of IL-6, STAT3, and NF-κB, and the resultant convergent trio-cascade may further deteriorate the barrier functionalities [68, 76] (Fig. 6).

Intracellular signaling mediated by STAT3 has been implicated in lung inflammation and in the pathogenesis of various lung diseases that affect men and women differentially [77, 78]. In this study, females displayed higher expression and/or phosphorylation of key elements of the IL-6R intracellular pathway, indicating that these mechanisms may mediate the observed increased inflammatory and cytokine gene expression previously reported by us in females vs. males [39]. In addition, our data indicate that the hormonal status of proestrus may predispose females to an increased inflammatory response to O_3_. Differential intracellular activation of JAK2/STAT3 and NF-κB/AKT1 pathways may be partially responsible for these effects (Fig. 6). In this regard, our study is the first to describe differential activation of these pathways in the lungs of male and female mice following acute O_3_ exposure, and the effects of hormonal status, as determined by the estrous cycle stage.

Studies have shown that O_3_, an air pollutant, can trigger allergic airway inflammation in women of reproductive age, and that these suffer more hospitalizations and death from asthma exacerbations than men [79, 80]. Since asthma complications occur at differential rates in women depending on their hormonal status (i.e., during the menstrual cycle, pregnancy, and menopause), and since these can be ameliorated with the use of oral contraceptives [20, 81, 82], it has been postulated that female sex hormones can act as physiological modulators of lung function and immunity in female patients. With the rise in the burden of inflammatory lung diseases in women worldwide, it is important to increase our knowledge of sex-specific mechanisms of immune response, as well as to understand the biological roles of sex hormones in modulating airway inflammation, innate immunity, and other processes relevant to the development and progression of these life-threatening conditions.

## Conclusions

In conclusion, our data indicate a sex-specific IL-6 mediated inflammatory response to acute O_3_ exposure via involvement of JAK2/STAT3 and AKT1/NF-κB pathways and variations of this response with the estrous cycle stage in females. Our results show that the female IL-6 mediated acute inflammatory response to inhaled O_3_ is higher in females than in males and that activation of specific intracellular mechanisms in this response in females is dependent of the hormonal status of the animal. Our work contributes to the overall understanding of the physiopathology of lung disease triggered by environmental exposures and hormonal status in women. Future studies will likely uncover points of intervention for lung disease therapies that will be specific for women and consider the hormonal status of the patient and facilitate the development of individualized medical treatments that are more efficient to treat lung inflammatory disease caused by air pollution.

## Additional file

Additional file 1: Figure S1. Analysis of (a) IL6, (b) IL6R, (c) STAT3, (d) STAT3-S727, (e) STAT3-Y705, (f) JAK2, (g) JAK2 phosphorylated (Y1007+Y1008), (h) NF-κB (p105/p50), and (i) AKT1 overall expression in animals exposed to FA and O_3_. Data expressed as Ranks-Kruskal-Wallis test of densitometric analysis; the values are depicted as mean with SD; here, ***p* ≤ 0.01 and *****p* ≤ 0.0001 are the levels of statistical significance compared to controls (*n* = 6–8 per group). (TIF 2773 kb)
