# Supplementary figures and images for: Sex-specific IL-6-associated signaling activation in ozone-induced lung inflammation
Source: Biol Sex Differ. 2016 Mar 5;7:16. doi: 10.1186/s13293-016-0069-7 (PMC4779258; doi:10.1186/s13293-016-0069-7)

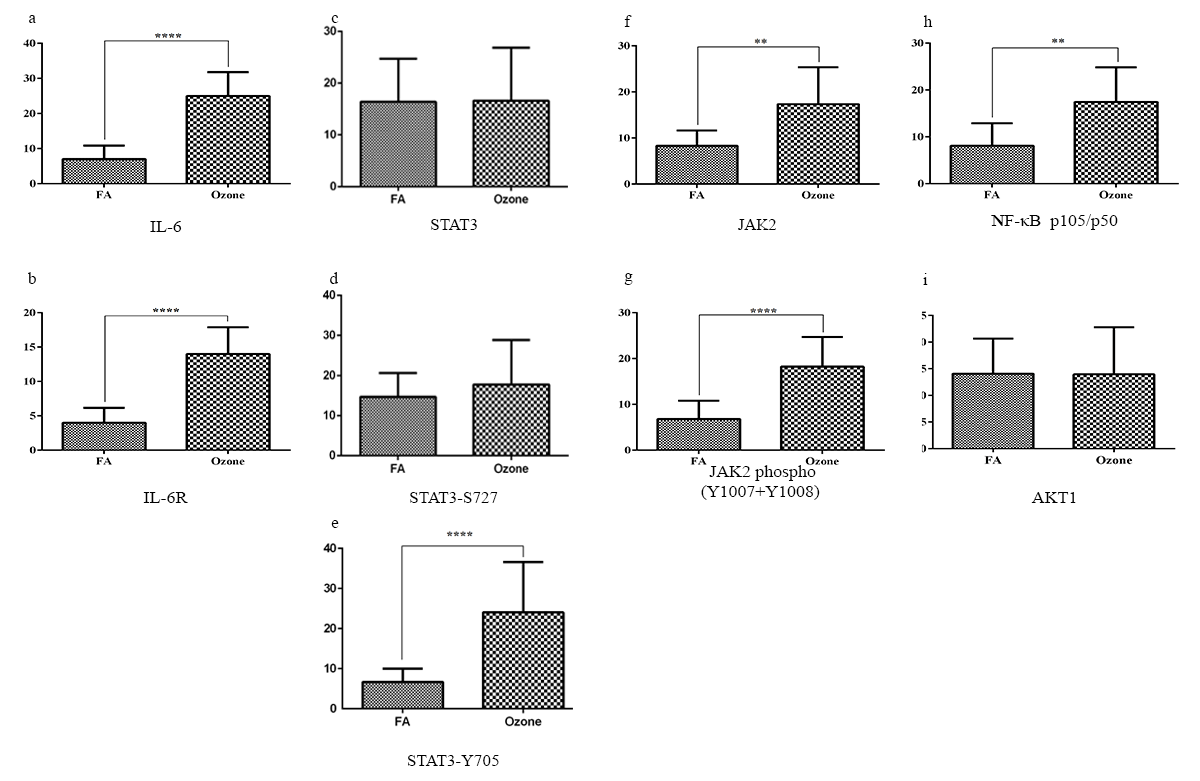

Supplement: Additional file 1: Figure S1. — Analysis of (a) IL6, (b) IL6R, (c) STAT3, (d) STAT3-S727, (e) STAT3-Y705, (f) JAK2, (g) JAK2 phosphorylated (Y1007+Y1008), (h) NF-κB (p105/p50), and (i) AKT1 overall expression in animals exposed to FA and O3. Data expressed as Ranks-Kruskal-Wallis test of densitometric analysis; the values are depicted as mean with SD; here, **p ≤ 0.01 and ****p ≤ 0.0001 are the levels of statistical significance compared to controls (n = 6–8 per group). (TIF 2773 kb) [file 13293_2016_69_MOESM1_ESM.tif]
